# Underwater Depth and Temperature Sensing Based on Fiber Optic Technology for Marine and Fresh Water Applications

**DOI:** 10.3390/s17061228

**Published:** 2017-05-27

**Authors:** Dinesh Babu Duraibabu, Gabriel Leen, Daniel Toal, Thomas Newe, Elfed Lewis, Gerard Dooly

**Affiliations:** 1 Optical Fibre Sensors Research Centre (OFSRC), University of Limerick, Limerick V94 T9PX, Ireland; Gabriel.Leen@ul.ie (G.L.); Thomas.Newe@ul.ie (T.N.); 2 Mobile and Marine Robotics Research Centre (MMRRC); University of Limeirck, Limerick V94 T9PX, Ireland; Daniel.Toal@ul.ie (D.T.); Gerard.Dooly@ul.ie (G.D.)

**Keywords:** temperature sensor, pressure sensor, optical fibre sensors, fibre bragg gratings, fabry-perot, ROV, ocean sensing, sub-sea temperature and pressure

## Abstract

Oceanic conditions play an important role in determining the effects of climate change and these effects can be monitored through the changes in the physical properties of sea water. In fact, Oceanographers use various probes for measuring the properties within the water column. CTDs (Conductivity, Temperature and Depth) provide profiles of physical and chemical parameters of the water column. A CTD device consists of Conductivity (C), Temperature (T) and Depth (D) probes to monitor the water column changes with respect to relative depth. An optical fibre-based point sensor used as a combined pressure (depth) and temperature sensor and the sensor system are described. Measurements accruing from underwater trials of a miniature sensor for pressure (depth) and temperature in the ocean and in fresh water are reported. The sensor exhibits excellent stability and its performance is shown to be comparable with the Sea-Bird Scientific commercial sensor: SBE9Plus.

## 1. Introduction

In the recent decades with the recognition of climate change and its potential effects for mankind, there has been significant attention given to the sensing of ocean parameters [1,2]. Oceanographers use various instruments for measuring the physical parameters of sea water which include conductivity (C), temperature (T) and depth (D) and such devices are generally referred to as CTD instruments. CTD instruments are evolving and are increasingly equipped with more accurate sensing capabilities [3]. Ocean CTD sensing is often undertaken using remote operated vehicles (ROV) or autonomous underwater vehicles (AUV), semi-autonomous vehicles, research vessels, underwater gliders, buoys [4] and oceanic predators [5,6]. The measurements taken using research vessels often provide high quality data but are limited in certain oceanographies due to the large size of such vessels. While sensors mounted on ROVs or AUVs provide information specific to the local region of interest, can be more flexible/controllable in terms of accessing specific locations, and are increasingly being used for environmental monitoring.

Many sensors for marine applications have been widely reported in recent years which are generally based on MEMS [4,7,8,9,10] e.g., resonant quartz crystal and piezo-resistive [11,12,13]. While optical sensors for pressure and temperature measurements have been used in various applications such as structural health monitoring, biomedical, oil and gas and in aerospace industries in recent years [14,15]. Advantages of optical fibre sensors include small in size; immune to electromagnetic interference; and relatively low cost; and can be utilized as a point sensor or a quasi distributed sensor [16]. Miniature sensors provide the opportunity to generally integrate easily into the CTD sensor networks which reduces the cost in the oceanographic studies [17]. The temperature (T) measurements are done as function of pressure or depth (D). As are salinity (S) measurements, which are more difficult and estimated indirectly via conductivity (C). A combination of several sensors with different characteristics is needed for CTD measurements. However, salinity measurement can be avoided by assuming a linear relationship between temperature and salinity (T–S relationship) which is valid for some ocean areas [18].

Several optical sensors for pressure and temperature measurement have been reported [19,20,21,22]. Here, we present a field trial of a combined optical fibre-based point sensor for monitoring pressure (depth) and temperature in underwater applications. The sensor is based on the combination of an extrinsic Fabry Perot interferometer (EFPI) and a Fibre Bragg Gratings (FBG) for measuring pressure (depth) and temperature respectively. The combined sensor is made entirely of glass, which provides excellent structural stability and is corrosion resistant. The FBG temperature sensing element is located in very close proximity to the pressure measurement element EFPI and serves two purposes: (a) temperature measurement at the measurement point; and (b) compensation of thermal cross sensitivity for EFPI sensor element. The packaging of the sensor itself and the supporting instrumentation used to perform the underwater evaluations in both sea water and fresh water is described.

## 2. Sensor Design

**Figure 1:**
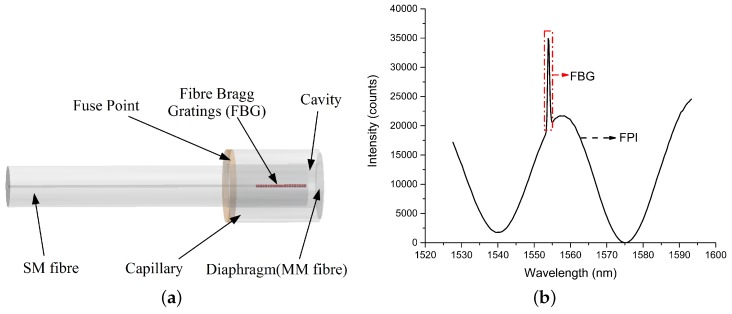
(**a**) Schematic of the Extrinsic Fabry Perot Interferometer (EFPI)/Fibre Bragg Gratings (FBG) sensor; (**b**) Reflection spectrum of the sensor showing EFPI and FBG.

A schematic of the EFPI pressure sensor is shown in Figure 1a [23]. A hollow core glass capillary with an outer and inner diameter of 200 μm and 130 μm diameter respectively is used. The capillary is spliced to a power core multimode (MM) fibre with an outer diameter of 200 μm using an Ericsson FSU 975 fusion splicer. A Corning single mode (SM) Fibre SMF-28 fibre with an outer diameter of 125 μm is inserted into the capillary. The capillary is collapsed on to the SM fiber using fusion splicer so as to leave an air gap of about 20–30 μm between the SM fiber’s end face and the previously fused MM fibre. The fabricated sensor is made completely from glass and the air cavity is completely sealed [24]. Thereafter, the MM fibre is cleaved and polished to a thickness of 10 μm in order to form a diaphragm. The diaphragm is then further reduced in thickness (up to 2–3 μm) by etching with hydrofluoric (HF) acid in order to ensure that the sensor’s desired operational range and resolution is linear. The complete diaphragm polishing and etching is monitored online using a LabVIEW*™* program developed by the authors by estimating the thickness theoretically, in order to ensure that the desired diaphragm thickness is achieved. The sensor diaphragm deflects when under pressure, and this deflection of the glass diaphragm can be accurately estimated using Youngs modulus (*E*) and the Poisson ratio (υ) of glass. A deflection of the diaphragm due to an external pressure changes (ΔP) changes the cavity length (ΔL), which in turn results in a wavelength shift of the optical spectrum returned from the sensor. The change in cavity length is given by the Equation (1) where *‘d’* is the thickness of the diaphragm and *‘a’* the radius of the diaphragm [25].

(1) $$\Delta L=\left(\frac{3(1-v^{2})}{16Ed^{3}}\right)\;\cdot \;a^{4}\;\cdot \;\Delta P=s_{p}\;\cdot \;\Delta P$$

The EFPI sensor spectrum is a combination of multiple reflected wave fronts interfering with each other. Referring to Equation (2), *I* is the intensity of the reflected signal, $\vec{E_{0}}$ is the electric field strength of the light reflected at the end-face of the SM fibre, $\vec{E_{1}}$ is the light reflected at the inner side of the MM fibre (diaphragm) and $\vec{E_{2}}$ for the light reflected at the outer side of the MM fibre (diaphragm).

(2) $$I={\left(\vec{E_{0}}+\vec{E_{1}}+\vec{E_{2}}\right)}^{2}$$

(3) $$I\left(\lambda \right)=E_{0}\;\cdot \;E_{1}\;\cdot \;\cos\left(\frac{4\pi n_{0}L}{\lambda }\right)+E_{0}\;\cdot \;E_{2}\;\cdot \;\cos\left(\frac{4\pi \left(n_{0}L+n_{1}d\right)}{\lambda }\right)+E_{1}\;\cdot \;E_{2}\;\cdot \;\cos\left(\frac{4\pi dn_{1}}{\lambda }\right)$$

where *‘L’* is the length of the cavity, ‘$n_{0}$’ and ‘$n_{1}$’ are the refractive indices of the air and diaphragm respectively, *‘d’* is the thickness of the diaphragm and ‘λ’ is the wavelength. The amplitude (intensity) of the reflected light wave is determined by the combination of the reflections from each interface. As noted earlier, the cavity length changes when the diaphragm deflects due to a change in the applied external pressure and thus modulates the light wave which leaves the sensor structure. An example of the reflected interference spectrum is shown in Figure 1b.

The fibre Bragg grating (FBG) temperature sensing element is fabricated by creating a periodic change of the refractive index in the core of the SM fibre. Light propagating through the SM FBG is partially reflected when the optical wavelength is equal to the Bragg wavelength $\lambda_{B}$. Several techniques have been widely reported for the fabrication of FBGs, such as exposing the fibre to a spatial pattern of ultraviolet light (UV) [26]; directly in the drawing tower during the fibre’s manufacture [27] and the authors have reported another novel technique which uses a femtosecond laser to inscribe the FBG when the SM fibre has been assembled into the EFPI element [28].

The Bragg wavelength depends on the effective refractive index ($n_{e}ff=1.447$) and the grating period (Λ). A change in temperature of the surrounding environment changes the spacing of the periodic gratings which in turn changes the reflected spectrum. The Bragg wavelength ($\lambda_{B}$) shifts linearly with a change in temperature (ΔT) and is given by the Equation (4) where ’*k*’ is the temperature sensitivity and $\lambda_{B}\left(T_{0}\right)$ is the initial wavelength at a given temperature $T_{0}$.

(4) $$\begin{array}{c} \lambda_{B}\left(\Delta T\right)=\lambda_{B}\left(T_{0}\right)+k\;\cdot \;\Delta T \end{array}$$

The optical fibre pressure and temperature sensor (OFPTS) has been fabricated to measure the pressure and temperature at a single point of measurement. The combination of EFPI/FBG results in the capability of simultaneous measurement of both pressure and temperature and can be expressed as a matrix, see Equation (5). The matrix can be manipulated such that the EFPI thermal cross-sensitivity can be compensated for, through the use of the FBG’s temperature measurement information. The inscribed FBG is completely (strain) relieved and is therefore insensitive to pressure changes. While the temperature sensitivity ($s_{t}$) of the EFPI pressure sensing element depends on the individual properties of the sensor once fabricated and can be determined empirically in advance of deployment [23].

(5) $$\left[\begin{array}{c} {\Delta \lambda }_{B}\\ \Delta L \end{array}\right]=\left[\begin{array}{cc} 0 & k\\ s_{p} & s_{t} \end{array}\right]\left[\begin{array}{c} \Delta P\\ \Delta T \end{array}\right]$$

## 3. Experimental Setup

### 3.1. Optical Setup

**Figure 2:**
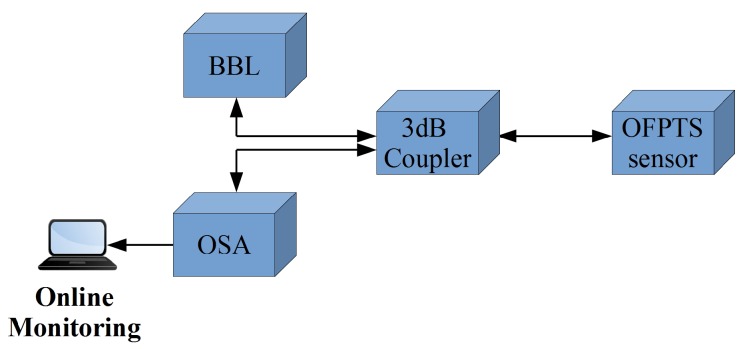
Schematic of the Optical Setup.

A schematic of the optical interrogation setup is shown in Figure 2. The interrogation system consists of a broadband light source (BBL) from Exalos (EXS210069-01, Exalos AG, Schlieren, Switzerland), a 3 dB coupler and an optical spectrum analyser (OSA) from Ibsen Photonics I-MON 512E (Ibsen Photonics A/S, Farum, Denmark). The broadband light source has a Gaussian output with a bandwidth of 45 nm centered at 1550 nm and an optical power output of 15 mW. The light propagates from the BBL source via the 3 dB coupler to the sensor, where it is modulated by the EFPI and FBG. The modulated light is reflected back by the EFPI and FBG and travels back down the SM fibre to the 3 dB coupler which channels the reflected light to the optical spectrum analyser (OSA). The OSA is based on a linear InGaAs image sensor from Hamamatsu Photonics and has 512 pixels with a wavelength fit resolution of <0.5 pm over the wavelength range from 1510 nm to 1595 nm. The OSA is connected by USB 2.0 to a PC, which runs a custom LabVIEW*™* application to interpret the signal information.

### 3.2. Marine Sensor Deployment in Sea Water

**Figure 3:**
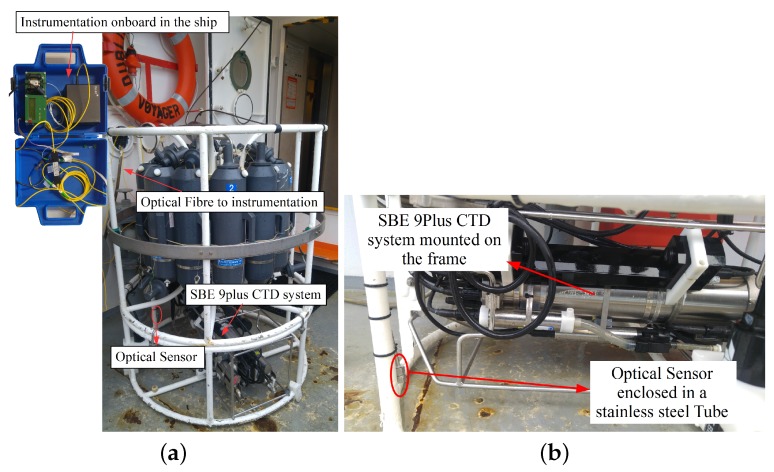
(**a**) Optical sensor mounted on the Conductivity, Temperature and Depth (CTD) rosette; (**b**) Sensor location with respect to the reference sensor.

The optical fibre sensor was packaged in a 316 stainless steel tube with one open end to allow contact with the surrounding water. Several holes were made in the circumference of the stainless steel tube to allow air to escape when the sensor was immersed in the water while the other end of the tube was sealed with a marine grade epoxy (Loctite${}^{®}$ Epoxy Marine) to hold the fibre in place. The OFPTS sensor was placed on the CTD rosette in reasonably close proximity to the reference sensor SBE 9Plus (Sea-Bird Scientific) as shown in the Figure 3b. The sensor was interrogated from the ship using the architecture illustrated in Figure 2, while Figure 3a shows the ship side interrogation system in a blue carry case. The sensor was deployed in the sea for the measurements and the GPS (Global Positioning System) location of the deployment locations was Location 1: NMEA (National Marine Electronics Association) Latitude = 51 49.54 N, NMEA Longitude = 008 16.31 W; Location 2: NMEA Latitude = 51 49.67 N, NMEA Longitude = 008 16.37 W. The measurement results are discussed later in Section 4.1.

### 3.3. Marine Sensor Bottle on ROV in Fresh Water

**Figure 4:**
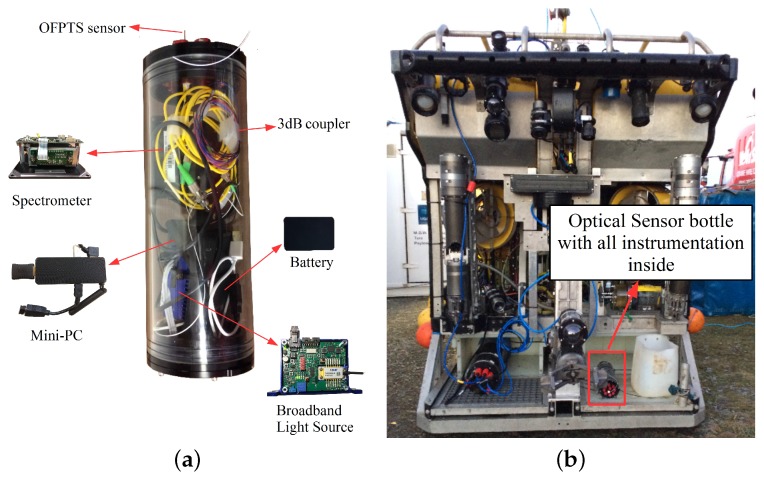
(**a**) Marinised bottle with all instrumentation inside; (**b**) Marinised bottle mounted on the Remote Operated Vehicle (ROV).

**Figure 5:**
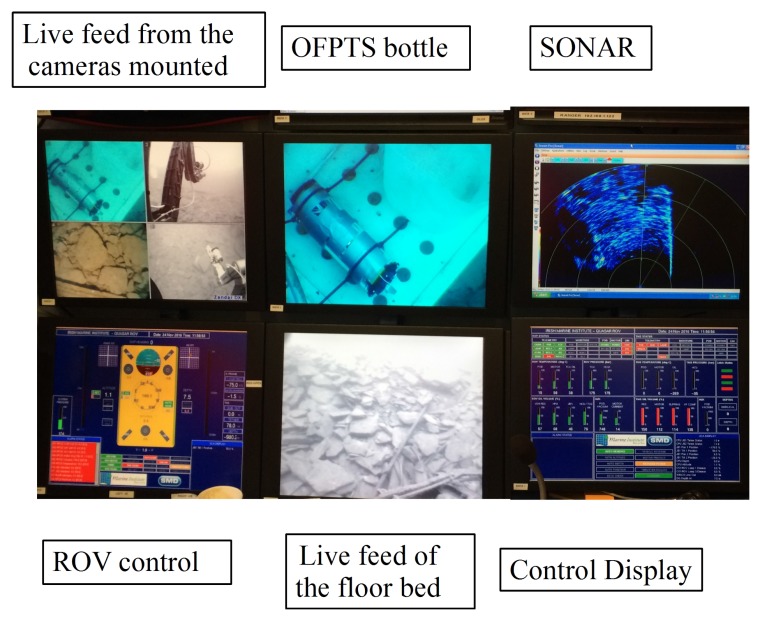
ROV (Holland1) on the floor bed which was controlled from the control station.

A schematic of the marine bottle with all the instrumentation for the optical sensor interrogation is shown in Figure 4a. The 4 in series bottle (BlueRobotics) is made from cast acrylic with a length of 11.75 in, with inner and outer diameters of 4 in and 4.5 in respectively. The bottle was sealed using aluminium end caps on both sides, with one end having provision for up to 10 cables to enter/exit the bottle. The bottle itself was rated to a maximum pressure of 1000 kPa (10 bar). The sensor was fed through one of the end cable glands and sealed with a marine grade epoxy (Loctite${}^{®}$ Epoxy Marine, Henkel Corporation, CT, USA), while the end plates on each end of the acrylic bottle cylinder were held tightly in place with stainless steel screws. A PC stick from NEXXT powered by a portable battery (10,000 mAh) was also included with the optical instrumentation in the bottle for data acquisition and had an estimated run time of 8 h. Figure 2 illustrates the contents of the marinised bottle: BBL source; optical spectrum analyser; PC stick; battery; and the 3 dB coupler. The marinised bottle and instrumentation was then mounted on the ROV (Holland1) as shown in Figure 4b and the control display for the ROV is shown in Figure 5.

## 4. Results and Discussion

### 4.1. Sensor Mounted on the CTD Rosette for Sea Water Deployment

**Figure 6:**
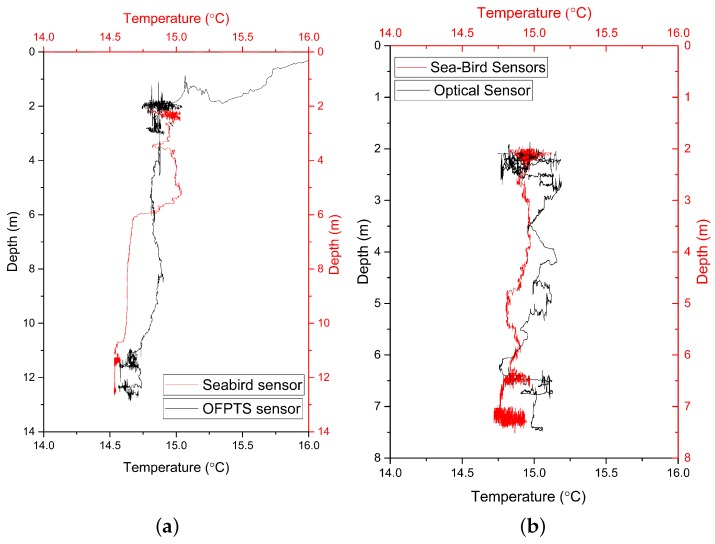
Depth and Temperature graph in comparison to the reference sensor (**a**) Deployment station NMEA (National Marine Electronics Association) Latitude = 51 49.54 N, NMEA Longitude = 008 16.31 W (**b**) Deployment station NMEA Latitude = 51 49.67 N, NMEA Longitude = 008 16.37 W.

**Figure 7:**
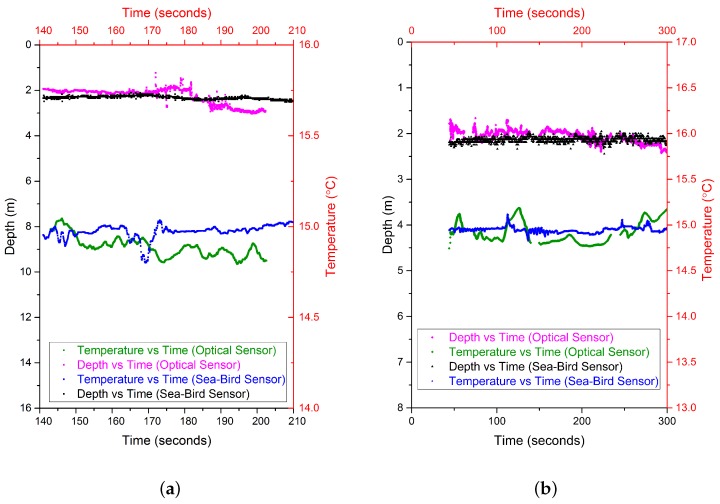
Depth, Temperature response vs time in comparison to the reference sensor (**a**) Deployment station NMEA Latitude = 51 49.54 N, NMEA Longitude = 008 16.31 W (**b**) Deployment station NMEA Latitude = 51 49.67 N, NMEA Longitude = 008 16.37 W.

Before deployment on the CTD rosette for sea water measurements, the EFPI pressure sensor was calibrated and its sensitivity and resolution determined to be 15 nm/kPa and 0.8 cmH${}_{2}$O respectively. The FBG temperature sensor was also calibrated and its sensitivity determined to be 12.5 pm/K with a resolution of 0.1 ${}^{\circ }C$ [29]. The OFPTS sensor was then mounted on a CTD rosette frame on the opposite side of the frame to the reference sensor and approximately at the same height as the reference sensor as illustrated in Figure 3. Figure 6a,b shows the depth and temperature response of the sensor at two different deployment locations. As can be seen from Figure 6, the optical and reference sensors exhibit a similar response with regard to the depth measurement. There is small difference in the response of the temperature sensors (<0.5 ${}^{\circ }C$) and this is attributed to minor localised differences in the water temperature between the two sensor locations. The crossing of the temperature at 6 m was noticed at the reference sensor in Figure 6a which is due to the oceanic mixed layer. This phenomenon can be attributed to the seasonal thermocline which occurs at shallow depths and is dependent on the season. The CTD rosette was initially deployed to 2 m and was held at this depth for some time before it was allowed to descend to deeper holding positions, as shown in Figure 7. The sensor’s temperature and pressure (depth) response versus time is shown in Figure 7a for deployment location 1 which had a holding depth of approximately 2 m. Figure 7b illustrates the sensor’s temperature and pressure (depth) response at Location 2 where the holding depth was again approximately 2 m.

### 4.2. Sensor Mounted on the ROV for Fresh Water Deployment

**Figure 8:**
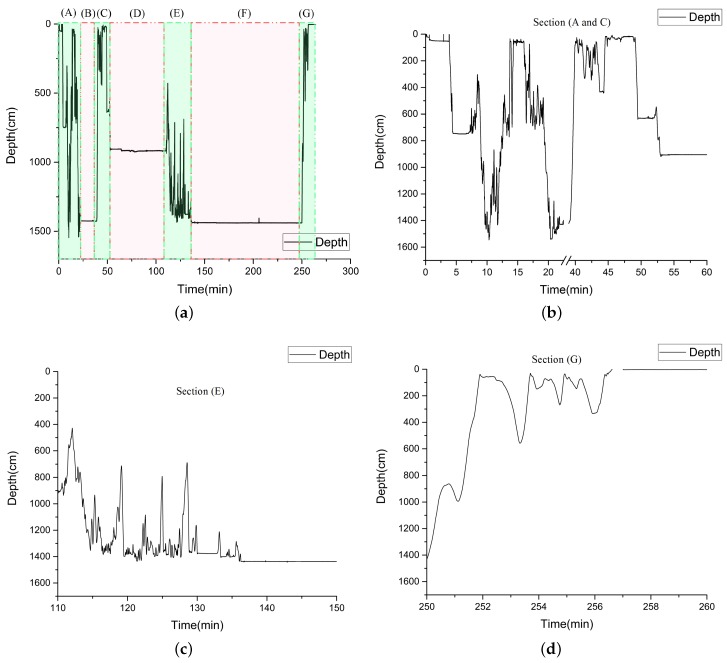

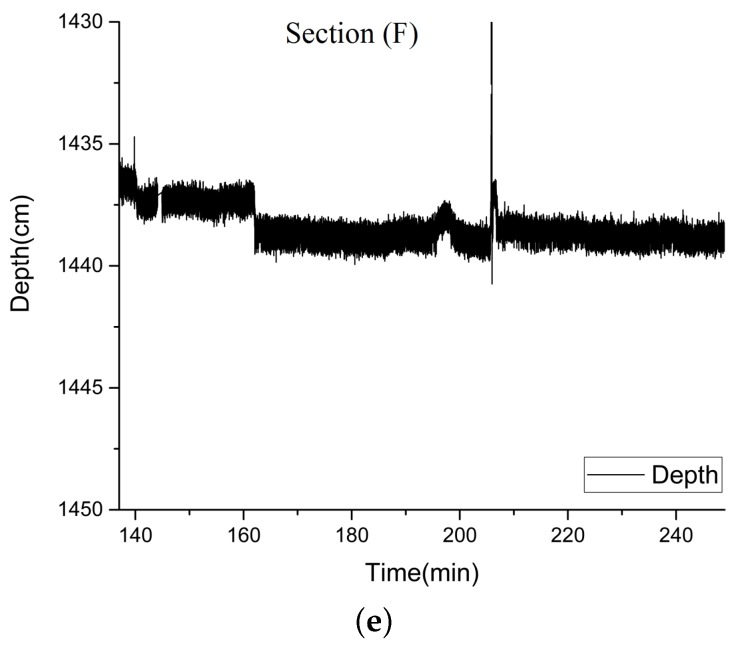
(**a**) Depth response of the sensor during the measurement over 4 h, (**b**) Depth response of the sensor in the sections (A) and (C) from (**a**), (**c**) Depth response of the sensor in the section (E) from (**a**), (**d**) Depth response of the sensor in the section (G) from (**a**), (**e**) Depth response of the sensor on the floor in the section (F) from (**a**).

The OFPTS and encapsulated instrumentation was mounted on the ROV as shown in Figure 4b. The ROV was deployed in a fresh water lake at an old slate quarry near Killaoe in County Tipperary, Ireland, where the water depth ranged from 7 m to 40 m. The reference sensors (depth and temperature) mounted on the ROV were calibrated for measurements in sea water which created unanticipated issues with the reference sensor data, hence it cannot be provided here as a reliable reference. Figure 8, shows the depth response of the OFPTS pressure sensor during the deployment. Figure 8a shows the time resolved sensor output for the entire 4 h period, during which time the ROV was also performing other measurements and manoeuvres To aid in the discussion and explanation, the response of the OFPTS sensor over the 4 h period is divided into a number of time intervals in Figure 8.

**Figure 9:**
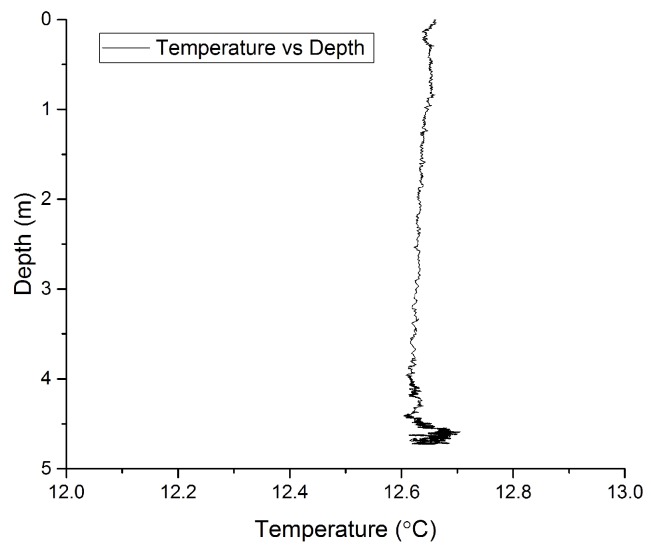
Depth and temperature measurement during deployment of the ROV (Holland1).

Figure 8b shows time intervals (A) and (C) of the 4 h deployment, when the ROV was relatively frequently altering its depth. Section (B) of Figure 8a graphs a period of time when the ROV was stationary at the bottom of the lake. Section (E), also shown in detail in Figure 8c, corresponds to a period of time when the ROV moved from a particular test location to the bottom of the lake and remained there for a longer time. Figure 8d corresponds to Section (G), and illustrates the depth measurement recorded when the ROV moved from the floor of the lake to the surface before being lifted out of the water. It is worth noting that the spikes in the measurement graph represent actual movement of the ROV and are not measurement noise, as during the measurement period the ROV was performing additional measurements and manoeuvres and furthermore the floor bed of the lake had an uneven surface which made it difficult to land the ROV at a precise location of choice. Figure 8e shows the sensor’s stability with an accuracy of about 2.5 cm when the ROV was on the floor. Figure 9 shows the temperature versus depth measurements recorded during the deployment. It was noted during the measurements that when the ROV was holding depth at approximately 4.5 m yet moving in the water at this set depth, a variation in the temperature was observed, as can be seen in the portion of Figure 9 which graphs the response for depths greater than 4 m.

The sensor was used in both sea water and fresh water to validate the sensor’s performance. The sensor measurements exhibited very good correlation with the reference ship based systems. Oceanographers use a variety of sensing techniques to monitor the physical parameters of the water column including measurements from the ship, ROV or AUV, gliders and buoys. There are significant advantages and disadvantages from the measurements of both ship based and autonomous instruments which compliment each other. The developed system is potentially well suited to a variety of marine-based application fields, e.g., Tsunami warning systems, wave and tide gauges, sub-sea vehicles, motion reference systems and construction/oil platform levelling. The calibration of industrial sensors in autonomous instruments is the main disadvantage since each of the sensors has to be calibrated individually and sometimes the historical data of the location is matched to the sensors. The use of historical data to assume the water properties pose a potential threat in missing changes in the water column. The ship-based measurements are also required in order to monitor the changes in the ocean [30].

## 5. Conclusions

In this paper, the results of underwater trials of the optical fibre-based pressure and temperature sensor and the instrumentation use in a fresh water and in a sea water deployment have been presented and discussed. The novel optical fibre sensor allows for the simultaneous measurement of pressure and temperature at a single location and has been developed for low power consumption, therefore being well suited to long-term deployments. The sensor’s pressure measurement resolution and its sensitivity were determined during the calibration to be 0.8 cmH${}_{2}$O and 15 nm/kPa respectively. The temperature sensor’s sensitivity was determined during calibration to be 12.5 pm/K which equated to a resolution of 0.1 ${}^{\circ }C$. The optical sensors exhibited a good correlation with a commercial reference sensor during the sea water deployment. To the best of our knowledge, this is the first time that the simultaneous measurement of temperature and depth in real time in both sea water and fresh water has been undertaken using an optical fiber based sensor.

## Abbreviations

The following abbreviations are used in this manuscript:

AUV	Autonomous Underwater Vehicle
CTD	Conductivity, Temperature and Depth
EFPI	Extrinsic Fabry-Perot Interferometer
EPSPG	Enterprise Partnership Scheme PostGraduate
FBG	Fibre Bragg Gratings
GPS	Global Positioning System
HF	HydroFluoric Acid
MM	MultiMode
NMEA	National Marine Electronics Association
OFPTS	Optical Fibre Pressure and Temperature Sensor
OSA	Optical Spectrum Analyzer
ROV	Remote Operated Vehicle
SM	Single Mode

## References

[1] Ship-based Repeat Hydrography: A Strategy for a Sustained Global Programme. http://unesdoc.unesco.org/images/0018/001865/186560e.pdf.

[2] Sea-Level Rise and Variability–A Summary for Policy Makers. http://unesdoc.unesco.org/images/0018/001893/189369e.pdf.

[3] Oldfield S. Pressure or depth?. Proceedings of the OCEANS’94.

[4] Aravamudhan S., Bhat S., Bethala B., Bhansali S., Langebrake L. MEMS based Conductivity-Temperature-Depth (CTD) Sensor for Harsh Oceanic Environment. Proceedings of the OCEANS 2005.

[5] Hooker S.K., Boyd I.L. (2003). Salinity sensors on seals: Use of marine predators to carry CTD data loggers. Deep Sea Res. Part I Oceanogr. Res. Pap..

[6] Boehlert G.W., Costa D.P., Crocker D.E., Green P., O’Brien T., Levitus S., Le Boeuf B.J. (2001). Autonomous pinniped enviromental samplers: Using instrumented animals as oceanographic data collectors. J. Atmos. Ocean. Technol..

[7] Broadbent H.A., Ivanov S.Z., Fries D.P. (2007). A miniature, low cost CTD system for coastal salinity measurements. Meas. Sci. Technol..

[8] Houston M., Paros J. High accuracy pressure instrumentation for underwater applications. Proceedings of the 1998 International Symposium on Underwater Technology.

[9] Mohan A., Malshe A., Aravamudhan S., Bhansali S. Piezoresistive MEMS pressure sensor and packaging for harsh oceanic environment. Proceedings of the 54th Electronic Components and Technology Conference (IEEE Cat. No. 04CH37546).

[10] Aravamudhan S., Bhansali S. (2008). Reinforced piezoresistive pressure sensor for ocean depth measurements. Sens. Actuators A Phys..

[11] Sea-Bird Scientific SBE9 Plus Manual. http://www.seabird.com/sites/default/files/ocuments/9plus_018.pdf.

[12] Ocean Seven 316 Plus CTD for Oceanography. http://www.idronaut.it/cms/view/products/multiparameter-ctds/environmental-ctds/ocean-even-316iplusi/s300.

[13] Valeport Ltd. miniCTD Data Sheet. http://www.valeport.co.uk/Portals/0/Docs/Datasheets/Valeport-miniCTD.pdf.

[14] Kersey A.D. (2000). Optical fiber sensors for permanent downwell monitoring applications in the oil and gas industry. IEICE Trans. Electron..

[15] Willsch R., Ecke W., Bartelt H. Optical fiber grating sensor networks and their application in electric power facilities, aerospace and geotechnical engineering. Proceedings of the 15th Optical Fiber Sensors Conference Technical Digest. OFS 2002 (Cat. No.02EX533).

[16] Grattan K., Sun T. (2000). Fiber optic sensor technology: An overview. Sens. Actuators A Phys..

[17] Crescentini M., Bennati M., Tartagni M. (2012). Design of integrated and autonomous conductivity–temperature–depth (CTD) sensors. AEU Int. J. Electron. Commun..

[18] Van Haren H., Gostiaux L. (2014). Characterizing turbulent overturns in CTD-data. Dyn. Atmos. Oceans.

[19] Lin C.M., Liu Y.C., Liu W.F., Fu M.Y., Sheng H.J., Bor S.S., Tien C.L. (2006). High-sensitivity simultaneous pressure and temperature sensor using a superstructure fiber grating. IEEE Sens. J..

[20] Yu Q., Zhou X. (2011). Pressure sensor based on the fiber-optic extrinsic Fabry-Perot interferometer. Photonic Sens..

[21] Bae H., Yu M. (2012). Miniature Fabry-Perot pressure sensor created by using UV-molding process with an optical fiber based mold. Opt. Express.

[22] Pevec S., Donlagic D. (2012). Miniature all-fiber Fabry–Perot sensor for simultaneous measurement of pressure and temperature. Appl. Opt..

[23] Bremer K., Lewis E., Moss B., Leen G., Lochmann S., Mueller I. (2009). Conception and preliminary evaluation of an optical fibre sensor for simultaneous measurement of pressure and temperature. J. Phys. Conf. Ser..

[24] Pinet E., Cibula E., Donlagic D. (2007). Ultra-miniature all-glass Fabry-Perot pressure sensor manufactured at the tip of a multimode optical fiber. Fiber Opt. Sens. Appl..

[25] Giovanni D. (1982). Flat and Corrugated Diaphragm Design Handbook.

[26] Rao Y.J. (1997). In-fibre Bragg grating sensors. Meas. Sci. Technol..

[27] FBGS Technologies GMBH DTG TECHNOLOGIES. http://www.fbgs.com/technology/dtg-technology/.

[28] Poeggel S., Duraibabu D., Lacraz A., Kalli K., Tosi D., Leen G., Lewis E. (2016). Femtosecond-Laser-Based Inscription Technique for Post-Fiber-Bragg Grating Inscription in an Extrinsic Fabry–Perot Interferometer Pressure Sensor. IEEE Sens. J..

[29] Duraibabu D.B., Poeggel S., Omerdic E., Capocci R., Lewis E., Newe T., Leen G., Toal D., Dooly G. (2017). An Optical Fibre Depth (Pressure) Sensor for Remote Operated Vehicles in Underwater Applications. Sensors.

[30] Steele J.H., Thorpe S.A., Turekian K.K. (2009). Measurement Techniques, Sensors and Platforms–A Derivative of Encyclopedia of Ocean Sciences.

